# Learning from the experiences of pregnant women participating in a research study investigating human cytomegalovirus shedding: A qualitative study

**DOI:** 10.1371/journal.pone.0292134

**Published:** 2023-11-30

**Authors:** Shari Sapuan, Paul T. Heath, Blair L. Strang, Asma Khalil, Christine E. Jones

**Affiliations:** 1 Centre for Neonatal and Paediatric Infection, St George’s, University of London, London, United Kingdom; 2 St George’s University Hospitals NHS Foundation Trust, London, United Kingdom; 3 Infection and Immunity, St George’s, University of London, London, United Kingdom; 4 Faculty of Medicine and Institute for Life Sciences, University of Southampton, Southampton, United Kingdom; 5 NIHR Southampton Clinical Research Facility and NIHR Southampton Biomedical Research Centre, University Hospital Southampton NHS Foundation Trust, Southampton, United Kingdom; University of St Andrews, UNITED KINGDOM

## Abstract

Transmission of human cytomegalovirus (CMV), from a pregnant woman to her fetus can cause congenital CMV infection, with life-long problems in some infected children. The presence of CMV in an infected individual’s bodily fluid is known as shedding. An individual can become infected with CMV through contact with another individual who is shedding CMV in their bodily fluid, and the avoidance of contact with infected fluids may reduce the risk of infection. We explored the experiences of pregnant women taking part in a study investigating CMV shedding, to identify the potential facilitators and barriers towards engaging pregnant women with CMV risk-reduction measures. Twenty pregnant women participated in semi-structured, end-of-study, telephone interviews, analysed using thematic analysis. They participated in an observational study investigating CMV shedding in pregnant women previously infected with CMV living with young children. Participating women considered that CMV testing of themselves and their newborns was a benefit of participation, without raising additional concerns. They identified that their participation was contingent on a balance of convenience and inconvenience, and benefits and risks. Participation increased their awareness of their hygiene-based practices, leading to behavioural modifications that put them in contact with urine and saliva of their children without instructions to do so. These behavioural modifications might interfere with household routines. However, they recognised it to be a time-limited risk-reduction measure, and felt empowered by the knowledge they had gained through study participation and the support they had received from their partners. Participating women gained an increased awareness of their behaviour, resulting in behavioural modification without instructions to do this, in line with previous findings that trial participation can impact on participants’ thinking about their behaviour with a possibility to influence change. Maternal research and risk-reduction measures should be centred around being informative, convenient, empowering, and supportive.

## Introduction

After primary infection with human cytomegalovirus (CMV), CMV establishes life-long latency in the infected human [1]. Non-primary infection can occur in an individual with pre-existing immunity to CMV (being CMV seropositive) if there is reactivation of primary infection, or if re-infection occurs with a different CMV strain [1]. Primary or non-primary CMV infection can result in excretion of CMV in an infected individual’s bodily fluids, such as (but not limited to) saliva and urine [1, 2]. The presence of CMV in a bodily fluid, which may be detected by culture techniques or through CMV deoxyribonucleic acid detection by polymerase chain reaction, is known as shedding [2]. An individual can become infected with CMV through contact with bodily fluids of another individual who is shedding CMV [1, 3]. Young children may shed CMV for a prolonged period, making this age group an important source of CMV horizontal transmission to other children, adults, and importantly, pregnant women [1].

CMV infection is usually associated with no symptoms, or only mild symptoms, in most healthy individuals, including pregnant women [1, 3]. However, when CMV infection is contracted in a fetus before birth by vertical transmission from the mother, it can cause congenital CMV (cCMV) infection, which can be associated with severe disease in some infants [1, 3]. Up to 25% of children with cCMV infection will have life-long problems, such as sensorineural hearing loss (the most common adverse sequalae), neurodisability, and visual impairment [4–6]. Despite the burden of adverse health outcomes associated with cCMV infection, most pregnant women are not aware of CMV and cCMV infection, and advice about it is not routinely provided as part of antenatal care in the United Kingdom [7–9]. A pregnant woman may reduce her risk of acquiring CMV infection by the avoidance of direct mucosal contact with a young child’s saliva and urine, such as not kissing directly on the lips, not sharing food and drink with them and hand washing after contact with saliva or urine [10–13]. There is evidence to suggest that this might be achieved through educational interventions [14–17].

This study aimed to explore the experiences of those participating in a research study investigating CMV shedding during pregnancy in CMV seropositive women with young children, and the potential facilitators and barriers towards engaging pregnant women in CMV antenatal education and behavioural change measures.

## Materials and methods

This qualitative interview study was nested within an observational study that was conducted to understand the feasibility of running an observational cohort study on CMV shedding during pregnancy in CMV seropositive pregnant women living with at least one child under the age of four years (*Cytomegalovirus Shedding Characteristics in Pregnant Women* (the cCHIPS study); Clinicaltrials.gov identifier NCT04021628). Eligible pregnant women were identified during routine antenatal appointments at a tertiary hospital in London, UK, and recruited between 9^th^ April 2019 and 30th December 2020. As part of the observational study, blood samples from all participating women were tested to determine their CMV serostatus. CMV seropositive women were enrolled, and serial saliva, urine, vaginal secretions, and blood samples were collected to assess for CMV shedding. A serial questionnaire was completed by the participating women to assess for hygiene-related behaviours. Women were aware that they were participating in a study about CMV, but were not given specific instructions about ways to reduce the risk of acquiring new CMV infection during pregnancy.

At the end of participation in the *cCHIPS* study, individual, semi-structured interviews were conducted to evaluate the participants’ experiences of study participation, on up to 20 participating women. The interview guide (Box 1) was developed collaboratively by the research team, consisting of open and closed questions with prompts. The series of questions were divided into two categories: the first was to explore the participants’ experiences of study participation (described as ‘direct’ experiences), and the second was to evaluate any subsequent non-specifically directed consequences of study participation (described as ‘indirect’ experiences).

**Box 1**

Interview guide. It consisted of open and closed-ended questions, divided into two categories: the first was to explore the participants’ experiences on their participation in the cCHIPS study (Clinicaltrials.gov identifier NCT04021628) focusing on the specific study procedures, and the second was to evaluate any subsequent non-specifically directed consequences of study participation.

We will ask you some questions about your experience of being in the study and how you think it could be improved.

First, we would like to ask you about your ‘direct’ experience of participating in the study:

- *Why did you decide to participate in the study*?
- *What did you like about the study*?
- *What did you learn by participating in the study*?
- *What (if anything) did you find inconvenient or difficult*?
- *Did you feel comfortable being approached for the first time about the study in the combined screening clinic*? *Did you feel you had enough information about the study before agreeing to have your blood screened for CMV*? *Is there any other information you would have liked at this stage*?
- *Did you discuss your participation with someone else before you decided to participate*?
- *What was your experience of having to complete the Background Questionnaire at the beginning*?
- *What was your experience of having to complete the Contact Questionnaire at each study visit*? *Was having it emailed to you most convenient*? *Do you remember if you complete it at every stage*?
- *What was your experience of having to complete the Feedback Questionnaire at the end*? *Was having it emailed to you most convenient*?
- *What was your experience of having to do the self-sampling of saliva*, *urine and vaginal secretions*?
- *Was self-sampling instead of sampling by a study team most convenient*?
- *Did you give bloods as part of the study*? *What was your experience of this*?
- *How did you feel about having four study visits*?
- *How did you feel about the study timings*?
- *Is receiving a text reminder for the visits most convenient*?
- *How easy or difficult was it to contact the study team (if you did) at the time of delivery*?
- *How did you feel about your baby being offered testing for congenital CMV infection*? *Did your baby have the test*? *If so*, *how did you feel before and after the test*? *What about how your partner felt*?
- *As a result of participating in the study did you access more information about CMV*? *If so*, *where from*? *Did you find the information you access useful*?
- *What changes would you make in the way the study was conducted*?

Now, we would like to ask you about any ‘indirect experience’ of participating in the study:

- *Has your participation in the study led you to make any changes to your behaviours*? *To guide you with your answers*, *we will prompt each behaviour according to the study contact questionnaire*:
  - *I wash my hands with soap after changing a dirty (poo) nappy*
  - *I clean my hands with alcohol gel after changing a dirty (poo) nappy*
  - *I wash my hands with soap after changing a wet nappy (urine only)*
  - *I clean my hands with alcohol gel after changing a wet nappy (urine only)*
  - *I wash my hands with soap after wiping my child(ren)’s nose*
  - *I clean my hands with alcohol gel after wiping my chid(ren)’s nose*
  - *I put my child(ren)’s dummy in my mouth (for example*, *if fallen on floor)*
  - *I eat left-overs on my child(ren)’s plate*
  - *I share cutlery with my child(ren) to eat with after they have used it*
  - *I drink from my child(ren)’s cup or bottle after they have had a drink from it*
  - *I kiss my child(ren) on the lips*
  - *Any other behaviour*
- *If you did*, *why do you think you changed your behaviour*? *How easy was it to change these behaviours*? *What helped you*? *What did you find particularly difficult to change*? *What made it difficult*? *Are there ways in which this could be made less difficult*?
- *Did you discuss your behaviour change with your partner*? *If so*, *what changes did he/she make*? *How supportive was your partner/family*? *Did he/she find it easy/difficult to incorporate these changes*?
- *Did you discuss this with your family members or friends*? *How supportive were they*?
- *If your behaviours changed*, *have they become normal in your household*? *Which ones*? *If no*, *why*?

The same interviewer conducted the interviews by phone, which were audio-recorded and transcribed verbatim. The transcribed interview data was analysed following the six phases of Thematic Analysis described by Braun and Clarke [18]. Where extracted quotes by the participants were included in the report, these are written in italic font and signified by the double quotation marks, and any omitted material are signified by the symbol ’…’.

The study was ethically approved by the National Health Service (NHS) Health Research Authority and London Brent Research Ethics Committee (19/LO/0161). Informed written consent was obtained from all participants. Authors SS and PH had access to information that could identify individual participants during or after data collection.

## Results

Seventy-eight participating women were invited to take part in the interview via email, of which twenty responded and all agreed to it. Twenty pregnant women took part in the interviews, as described in the Methods section, with each interview lasting between 18 and 40 minutes. Each participating woman was interviewed once. Table 1 describes the demographic characteristics of women interviewed. The following two main themes were identified: 1) research participation is contingent on a balance of convenience and inconvenience, and benefits and risks, and 2) research participation in pregnancy is associated with subsequent non-specifically directed changes to usual behaviour influenced by awareness, knowledge, and support. The themes and subthemes that emerged from the 20 interviews are outlined in Table 2.

**Table 1:** Participant demographics.

Demographic Category	Demographic Details	Frequency (%; n = 20)
**Age**	30–34	7 (35%)
	35–39	11 (55%)
	40–44	2 (10%)
**Ethnicity**	White	17 (85%)
	Black/ African/ Caribbean/ Black British	0 (0%)
	Asian/Asian British	2 (10%)
	Mixed/multiple ethnic groups	1 (5%)
**Birth country in relation to UK**	Born in UK	14 (70%)
	Not born in UK	6 (30%)
**Length living in UK**	5–15 Years	6 (30%)
	>15 years	14 (70%)
**Education**	PhD or equivalent	1 (5%)
	Masters degree or equivalent	8 (40%)
	Undergraduate degree	8 (40%)
	Postgraduate certificate, diploma or equivalent	3 (15%)
**Number of pregnancies mean (SD)**		3 (1.07)
**Number of children aged <4 years**	1	20 (100%)
	>1	0 (0%)
**Living arrangement**	Living in a couple (irrespective of marital status)	20 (100%)
	Not living in a couple	0 (0%)
**Type of household members**	A cohabiting couple or single parent family (parents and children)	19 (95%)
	Multi-generational families (grandparents, parents and children)	1 (5%)

**Table 2:** Themes and sub-themes emerged from interviews with pregnant women.

Themes	Subthemes
Research participation in pregnancy is contingent on a balance of convenience and inconvenience, and benefits and risks	Research involvement should be convenient
	Additional health screening is a benefit
	The benefits of research procedures around the time of birth needs to be balanced against the risks of poor research compliance
	Collaboration between clinical and research teams needed to streamline research activities alongside routine care
	Research involvement should have the right balance of low intensity and high monitoring
Research participation in pregnancy is associated with subsequent non-specifically directed behavioural modification influenced by awareness and support	A reflection on behaviour can result in behavioural modification
	Behaviour is modified to reduce the risks of infection
	Feeling informed empowers behavioural modification
	Behavioural modification is a time-limited risk-reduction measure
	Behavioural modification is aided by prompts and partnership

### Theme 1: Research participation in pregnancy is contingent on a balance of convenience and inconvenience, and benefits and risks

Five subthemes were identified that relate to this overarching theme:

#### Subtheme 1.1: Research involvement in pregnancy should be convenient

The study procedures and study visits, as well as how the study was conducted (for example, the ability to self-sample, the ability to complete questionnaires via email in participants’ own time, study visits coinciding with routine antenatal appointments), were frequently described as “easy”, “convenient”, “flexible”, and “accommodating”. These positive experiences facilitated their participation.

“*I guess it was kind of something that happened whilst I was pregnant*, *and because I was at the hospital so much anyway*, *it just kind of happened alongside me being there*, *it wasn’t an inconvenience for me to do it*, *because I was there anyway doing the things I needed to do*, *and I was doing it in addition to that*.*”*

“*It definitely*, *it made it easier to keep on top of the appointments*, *that made it easier for everyone to keep track of…it couldn’t have been*, *I honestly don’t think it could have been any easier*, *everything*, *all my appointment took place when I would be waiting anyway…”*

#### Subtheme 1.2: Additional health screening is a benefit

Most participants felt that being informed about CMV infection, and the ability to find out their own CMV serostatus, were valuable and informative. Most participants and their partners also felt that the opportunity to have their newborns tested for cCMV infection was a benefit of participation. Most participants described that they had not put much thought into the outcome of the CMV test of their newborn during the study.

“*…the CMV thing*, *it’s not tested in women that are pregnant*, *that they’re not tested for it*, *and I think that they should be…once you’ve found out that you’ve got it*, *it might be too late*, *and there might be*, *like*, *bad effects to the baby*.*”*

“*…it’s fine because even if he has got something then it would be identified and he would be referred appropriately*, *as opposed to if I hadn’t taken part in the study*, *then not that it would have happened*…*”*

#### Subtheme 1.3: The benefits of research procedures around the time of birth should be balanced against the risks of poor research compliance

Some participants felt that study procedures, such as self-sampling of vaginal secretions or completing questionnaires, were challenging to perform around the time of birth. Some found the practicalities difficult, whilst others had trouble in remembering to perform the procedures.

“*I think again*, *just the last one*, *doing the vaginal swab at the last one*, *when I had the baby*, *probably not wise really*, *you don’t know what’s happened down there*, *and doing that one was really*, *I think I shouldn’t have done it really*. *I think you are in a bit of a daze*, *you don’t know what kind of damage and it’s uncomfortable*, *but I felt like I should do it*, *probably should have said no for that last one…”*

“*…actually*, *when my baby was born*, *that time*, *it’s really hard to give you the sample and everything*, *because you’re bleeding…most likely you’re in pain*, *and you forget as well*, *so I think it was like*, *how is it that I can remember*, *and give you everything*, *when you’re in so much pain*.*”*

#### Subtheme 1.4: Collaboration between clinical and research teams needed to streamline research activities alongside routine care

Some participants felt that even though having the study visits running alongside routine antenatal appointments was convenient, the lack of involvement by the clinical team with the study caused problems. This included the inability to provide their study samples to the clinical team, and the clinical team being unaware of the study. The participants felt that they were unable to discuss the study with the clinical team, which was challenging.

“*Sometimes it would be difficult to know when somebody would come to collect my samples*, *so it was always tied in as I said with my appointments*, *but you know no time was made with me a lot of the time*, *either it’s you know your time for the scan is on this day at this time*, *we will come and collect it at some point while you are at the hospital…I was always waiting for a phone call or I had to phone to try and find someone*, *so I’d say that was probably the only thing*, *I was never 100% sure if someone was actually going to come and collect it*.*”*

“*…for me as I said it was fine but the midwives*, *it all seemed like they didn’t know that it was going to happen*, *and it was all really quick; “oh is it ok if you take some extra blood for this”…the other midwife was just probably slightly taken aback*, *and wasn’t entirely clear with what she should be doing*, *and I would just say if that coordination could happen better*.*”*

#### Subtheme 1.5: Research involvement should have the right balance of low intensity and high monitoring

Although the study was designed to keep participation convenient, easy, and non-time consuming, some participants felt that to have a regular contact, reminder and refresher throughout the study would be beneficial. This is because they were pregnant as well as caring for a young child and could lose track of their place in the study journey, or even forget about the study altogether.

“*I think perhaps more regular sort of contact points would have been useful…sort of at the full front of your mind a bit more*, *as it’s over a long period of time*, *and you know if you’re pregnant and you’re working you’re just busy*, *and you’ve got another child*, *just to make it as easy as possible for the participants*, *I think the whole nudge theory*, *it’s never harmful to give people a gentle reminder before they have to do it*, *I think would be helpful*.*”*

“*…or like a quick sheet for the mother*, *as it did make me*, *“oh*, *you’re taking part in a survey*, *you don’t even know what it’s for*?*” and I just felt a little bit like*, *“oh yeah maybe I should”*, *I did know at one point*, *but you know what your memory is like when you’re pregnant…it would be like*, *as well as giving more information*, *a little bit more empowering to have it as an easy reference for*.*”*

### Theme 2: Research participation is associated with subsequent non-specifically directed behavioural modification influenced by awareness and support

Five subthemes were identified that relate to this overarching theme:

#### Subtheme 2.1: A reflection on behaviour can result in behavioural modification

Completion of the serial questionnaires to assess for hygiene-related behaviour with their children encouraged most of the participants to reflect upon their hygiene practices. In some, this resulted in a change to their behaviours during study participation.

“*I think you obviously sometimes feel like it’s a test don’t you*, *especially when it’s to do with hygiene and stuff*, *but they were absolutely fine*, *it kind of made me feel like*, *‘oh*, *should I be doing these things’*, *am I right or am I wrong*.*”*

“*I guess I was conscious when I was doing the form that because I was part of the study*, *I was being more careful about my behaviour and things*. *So I just felt like I was*, *I wasn’t doing any of the behaviours that you were asking about*. *And so then I wondered how*, *I don’t know*, *how that was affecting the results*.*”*

#### Subtheme 2.2: Behaviour is modified to reduce the risks of infection

Most participants’ decision to change their behaviour, was motivated by a desire to reduce the risks of spreading infection in general, not just of CMV infection specifically.

“*I think because I wanted to be more hygienic anyway*, *I carry tissue around to wipe her nose*, *the dummy thing I think that’s just a bad habit*, *I think it’s not very clean*, *so I just sort of clean it instead of sucking on it*, *I think because I was asked those questions it made me think I should do those definitely*.*”*

“*Generally thinking more about hygiene and thinking actually things can pass*, *thinking doing things like that you can spread it around*, *so instead of just rinsing with water use soap*, *I don’t know I guess that you live amongst germs so much and if you are part of a study*, *it makes you think about it more and you know*, *this may help in other area as well*.*”*

#### Subtheme 2.3: Feeling informed empowers behavioural modification

Most participants who made changes to their behaviour during their study participation, described them to be an easy adjustment because of the sense of feeling informed, empowering them to act on their reflections. Some participants even felt empowered to relax their hygiene practices during pregnancy because of their understanding that being CMV seropositive puts them at a lower risk of transmitting CMV infection to their unborn child, compared to primary CMV infection in pregnancy.

“*It was fine really*, *you know it was just something I wasn’t really consciously doing*, *and just being a bit more*, *thinking more about it*, *so yeah very easy*.*”*

“*Before it was something that I was conscious of*, *and that I was trying to follow guidelines to avoid contact but I did find it quite difficult*, *because when you have a toddler you do have contact all the time and they’re not the most hygienic creatures so either you just stop contact altogether or you kind of have to put up with that higher level of risk*, *but it’s quite anxiety-inducing if you do feel like you’re potentially risking your unborn child’s health*, *but you also don’t want to*, *you know*, *affect your existing child emotionally by seeming to reject them…I think guidelines aren’t very easy to follow in practice*, *which is why I sort of found it quite a relief to know that I wasn’t sort of at risk*, *to be slightly less cautious without feeling too guilty about it*.*”*

#### Subtheme 2.4: Behavioural modification is a time-limited risk-reduction measure

Some participants who made behavioural changes to their hygiene practices during pregnancy recognised that the behavioural modifications were to reduce the risks of CMV infection to their child before birth, which allowed them to revert to normal practices for them following delivery of the infant.

“*…because it’s easier*, *because I don’t have to be careful anymore because baby is born*, *so not a concern anymore for me on what you have to be careful with*, *you know all of this it was part of these*, *kind of*, *safety instructions while you’re pregnant*.*”*

“*But the other ones*, *I guess*, *because I’m probably unlikely to get pregnant again*. *So I guess I started like sharing a bottle and things like that*. *So we haven’t really kept them up because I feel like the risk is low*.*”*

#### Subtheme 2.5: Behavioural modification is aided by prompts and partnership

Some participants found it difficult to maintain the behavioural modifications, after they were no longer being prompted by the study questionnaire to assess for hygiene-related behaviour with their children. Some participants also found the behaviour changes they made during the study were difficult due to the impracticality and having to change their routine. These difficulties were eased through the support and reminders they received from their partners. Most participants who made behavioural changes involved their partners in their decision.

“*…my partner helped reminding me when he saw me doing it*.*”*

‘‘*Yes*. *I think he* (partner) *would explain it to my son as well*. *He would tell him that he couldn’t drink from my drink or tell him that he couldn’t share my food…we would explain why as well*.*”*

‘‘*Because my husband and I did it* (behavioural change), *it wasn’t hard*.*”*

“*Never easy to change a behaviour*. *I mean it comes and goes*, *it’s not necessarily conscious behaviour change*, *it’s just all of a sudden you sort of doing it again and then you don’t and then you think this is what I’ve got to do and you don’t think twice about it*.*”*

## Discussion

This qualitative study sought to explore the experiences of pregnant women taking part in an observational research study about CMV shedding in pregnancy and to identify the potential facilitators and barriers towards engaging pregnant women with CMV risk-reduction measures.

Our findings that convenience of participation is a key reason for pregnant mothers, especially those with young children to minimise any potential impact on childcare, to take part and remain in a study have reaffirmed that convenience should be at the core of study design. Paradoxically, low-intensity study involvement reduces engagement between the participant and the study team, which could impact on study compliance and retention. In future studies, ensuring that the design involves regular contacts, monitoring and reminders, whilst still maintaining convenience of study participation, would be beneficial to both the participants and research team.

A collaboration between the research and clinical teams is essential to ensure that research procedures and clinical appointments can run in synchrony, as also shown in previous research [19]. The timing of research involvement is another key consideration when designing a maternal study. It is important to weigh up the benefits for a pregnant woman to complete a study procedure around the time of birth against the risks of poor study compliance.

Antenatal screening for CMV infection and neonatal screening for cCMV infection are not part of routine maternity care in the UK. The fact that pregnant women valued the ability to know their own and their newborns’ CMV status without inducing unintended anxiety or concerns, is reassuring not only for future studies, but also for the potential implementation of routine antenatal and neonatal CMV screening. The concern of inducing anxiety in pregnant women through the acquisition of information on CMV without being able to provide solutions to treat it, has been recognised to be a factor in the reluctance of health care professionals to include CMV in routine antenatal education [8]. However, in line with our findings, the same research also found that pregnant women were keen to acquire knowledge about CMV and were motivated to reduce risks of CMV to their unborn child [8].

This was a non-interventional study, where no recommendations to perform hygiene-based behaviours were made, nor was information provided of any hygiene-based behaviours that could promote the risk of CMV infection to them or their unborn child. However, the serial questionnaires to assess the pregnant women’s hygiene-based behaviours used induced the reflection of and heightened awareness into their own behaviour, some enough to have resulted in a change to their behaviours during study participation. Our findings are in line with previous research that has highlighted that trial participation had an impact on the participants’ thinking about their own behaviour, with a possibility to influence change [20]. The potential for subsequent non-specifically directed changes of behaviour through study participation should be evaluated when designing a trial to consider its influence on the study results.

Knowledge of CMV serostatus empowered women participating in the study to make informed decisions on their behaviour. Most pregnant women modified their behaviour to reduce contact with saliva and urine of their young child, with the aim of reducing the risk of a new CMV infection which could be passed on to their unborn child. However, some did not adapt their behaviours because of the perception that the risk of vertical transmission was significantly reduced, as they already had CMV immunity. The ability of the knowledge of maternal CMV serostatus to have an impact on the perceptions to risk that may result in a behavioural change, may be an important consideration in the evaluation of antenatal CMV screening.

Our findings have highlighted that behavioural change messages about CMV in pregnancy should be framed as a short-term measure instead of a long-term measure to make it more attainable, and, in line with previous research [8], as a risk-reduction measure. We have also shown that behavioural change messages about CMV should be framed in the context of advice about other infections, to make behavioural change measures during pregnancy more achievable, relatable, and practical for women. Our study has also identified the value of involving partners in antenatal education on CMV, especially on the continual support they are able to provide to the pregnant women in implementing behavioural measures to reduce the risks of CMV. Moreover, our study has shown that continual prompts is a key factor to sustain behavioural change measures throughout pregnancy, which can be provided by their partners. Research also supports the inclusion of partners for behavioural change [8, 21].

Although the study was limited to 20 participating pregnant women, thematic saturation was achieved it provided rich data highlighting the experiences of participating women and reveals the potential facilitators and barriers towards engaging pregnant women in CMV antenatal education and risk-reduction measures. The lack of ethnic diversity may have had an impact on the findings and therefore warrants further investigation.

Finally, while the behavioural modification identified in our study was specific to pregnant women with existing children, most of the themes and subthemes identified from our study can be applied in most context of maternal research. We recommend for research involving pregnant women, especially in those with existing children, to be centred around convenience of participation. We also recommend it to be informative, empowering, holistic, and supportive.

This qualitative study provided a richer understanding of the pregnant mothers’ experiences and perspectives on participation in research. It showed that study participation during pregnancy in women with young children is feasible. It provided us with lessons to be learnt and a better understanding of the potential facilitators and barriers towards engaging pregnant women in CMV antenatal education and risk-reduction measures, which can be applied in other contexts of maternal research.
